# Role of Gluten-Free Diet on HbA1c Level in Children with Type 1 Diabetes Mellitus and Celiac Disease

**DOI:** 10.5812/ijem-144736

**Published:** 2024-09-29

**Authors:** Hedieh Saneifard, Ali Sheikhy, Aida Fallahzadeh, Marjan Shakiba, Maryam Kazemi Aghdam, Asieh Mosallanejad

**Affiliations:** 1 Mofid Children Hospital, Shahid Beheshti University of Medical Sciences, Tehran, Iran; 2 Non-communicable Disease Research Center, Endocrinology and Metabolism Population Sciences Institute, Tehran University of Medical Sciences, Tehran, Iran; 3 Cardiovascular Diseases Research Institute, Tehran Heart Center, Tehran University of Medical Sciences, Tehran, Iran; 4 Pediatric Pathology Research Center, Research Institute for Children's Health, Shahid Beheshti University of Medical Sciences, Tehran, Iran; 5 Shahid Beheshti University of Medical Sciences, Tehran, Iran

**Keywords:** Diabetes Mellitus Type 1, Celiac Disease, HbA1c, Anthropometric

## Abstract

**Background:**

Due to autoimmune mechanisms, celiac disease (CD) may affect patients with type 1 diabetes mellitus (T1DM) more than the general population.

**Objectives:**

We evaluated the effect of a gluten-free diet (GFD) on HbA1c levels in patients with both type 1 diabetes and CD.

**Methods:**

In this cross-sectional study, biochemical and clinical information was gathered from 174 children with T1DM from January 2013 to January 2019.

**Results:**

We assessed 174 children with T1DM (93 girls and 81 boys). Celiac disease was diagnosed in 18 out of 174 cases (10.34%). Height and weight percentiles showed significant differences between children with CD and those without CD (P = 0.015 and P = 0.026, respectively). The average HbA1c in the celiac group was 8.61 ± 2.20 (95% CI: 5.1 - 12.1) prior to GFD therapy. HbA1c was assessed six and twelve months following the initiation of the GFD and was found to be 8.32 ± 1.46 (95% CI: 6 - 9.8) and 8.37 ± 1.67 (95% CI: 6.1 - 10.2), respectively. No significant change in HbA1c was observed before and after therapy (P = 0.501).

**Conclusions:**

Diabetic children with CD exhibit lower weight and height compared to those without CD. Gluten-free diet therapy in patients with CD did not affect HbA1c levels.

## 1. Background

Celiac disease (CD) is an inflammatory disease affecting the small intestine, leading to the malabsorption of minerals, nutrients, and vitamins due to sensitivity to gluten in grains (1, 2). Studies show that both environmental and genetic factors are involved in the disease's pathogenesis (3-6). According to several studies, the prevalence of CD worldwide is nearly 1% (7-12). Common symptoms of CD include weight loss, growth retardation, anemia, osteoporosis, malabsorption, chronic constipation, and abdominal pain. However, some patients present with non-classic symptoms or may be asymptomatic (silent CD) (7). Early diagnosis and treatment in childhood are important to prevent short stature in adulthood (13, 14).

Type 1 diabetes mellitus (T1DM) is a chronic disease affecting children. It is classified as an autoimmune disease due to the destruction of β-cells, leading to absolute insulin deficiency (15-17). Due to shared genetic components and the autoimmune nature of these diseases, CD may occur more frequently in T1DM patients compared to the general population. A pooled analysis of 26,504 patients with T1DM found that the prevalence of CD was 6% in diabetic children, six times higher than the prevalence of CD alone (18). Another study reported the prevalence of CD in diabetic patients to be around 4%, with a higher prevalence in Asia (6.53%) compared to the USA (4.89%) (19).

In T1DM patients, CD often presents in atypical, silent, or potential forms (20). According to guidelines by the European Society for Pediatric Gastroenterology, Hepatology, and Nutrition (ESPGHAN), asymptomatic children and adolescents with an increased risk for CD, such as those with T1DM, should be tested for CD (21).

Limited data exist on HbA1c levels in adolescents with coexisting type 1 diabetes and CD. Some studies have reported higher HbA1c levels in patients with both type 1 diabetes and CD (22), while others have found no difference (23-25). Few studies have compared HbA1c in diabetic children and adolescents with CD undergoing gluten-free treatment (26).

## 2. Objectives

In this study, we evaluated the prevalence of CD in T1DM patients visiting a tertiary referral center for children in Tehran, Iran. We also analyzed their anthropometric indices and compared the occurrence of CD with the age of onset of diabetes. Additionally, we assessed the longitudinal changes in HbA1c levels in diabetic children with CD.

## 3. Methods

### 3.1. Research Design

In this single-center, cross-sectional study, we prospectively enrolled 174 patients with T1DM (diagnosed based on the American Diabetes Association (ADA 2015) criteria (27) and normal hemoglobin for age and sex (28) who were referred to a tertiary pediatric referral center in Tehran, Iran, between January 2013 and January 2019. The mean follow-up period was 24.2 months, equivalent to 351 person-years, with a minimum duration of follow-up of 18 months.

To evaluate these patients, we reviewed the records of diabetic patients admitted to the endocrinology ward and clinic. All children with T1DM were screened for CD at the time of diabetes diagnosis and every six months thereafter. Screening was conducted using serological testing by measuring serum IgA anti-tissue transglutaminase (anti-tTG) concentrations. Serum total IgA levels were also measured to detect IgA deficiency, and in cases of IgA deficiency, anti-tTG IgG was measured as an alternative. The samples were assessed for IgA anti-tTG using ELISA kits according to solid-phase enzyme immunoassays (CeliAK IgA LINE-4208, Generic Assay, Germany). If anti-tTG IgA was elevated, patients underwent intestinal biopsy for pathological examination. The inclusion criteria for patients undergoing intestinal biopsy were as follows: (1) positive anti-tTG IgA > 40 U/mLalone; (2) positive anti-tTG IgA with a lower titer (20 - 40 U/mL) in two consecutive readings over six months.

A pathologist reviewed the biopsies and reported the findings according to Marsh's classification. The Marsh grading system evaluates the presence of an immune reaction in the epithelium and reports the level of architectural alterations in the mucosa. The diagnosis of CD was established based on both clinical and pathological features in the seropositive group.

For each patient, we documented chronological age, height, weight, thyroid function test (TFT) results, and age at the onset of diabetes. Anthropometric indices in the celiac group were reported at the time of CD diagnosis, whereas they were reported based on the last visit for the non-celiac group. We also measured HbA1c levels before, six months after, and 12 months after gluten-free diet (GFD) treatment in the celiac group. Adherence to GFD treatment was monitored by periodically measuring celiac antibody levels and checking for the absence of clinical symptoms.

Children with hypothyroidism and CD were managed according to an accepted protocol. The Institutional Ethics Committee approved the research protocol. We calculated Body Mass Index (BMI), height-for-age, and weight-for-age percentiles in all cases using the CDC 2000 growth chart (3 - 97 percentile). Height was measured using a standard Secca Scale, with measurements taken while standing and recorded to the nearest 0.1 cm. Weight was measured using a digital scale (GS49, Beurer, Germany) with patients wearing light clothing. Body Mass Index was calculated using the Quetelet formula: Weight (kg) divided by height squared (m²).

In the celiac group, HbA1c was measured before celiac treatment and six and 12 months after GFD intervention. HbA1c levels were measured using high-performance liquid chromatography (HPLC) with a Labnovation LD-600 (China).

The Ethics Committee and Research Deputy of Shahid Beheshti University of Medical Sciences and Health Services approved this study (IR.SBMU.MSP.REC.1391.5), which was conducted in accordance with the Helsinki declaration. Written informed consent forms were obtained at the start of the study.

### 3.2. Statistical Analysis

The normality of the data distribution was assessed using the Shapiro-Wilk test. Continuous data were expressed as mean and standard deviation, while categorical data were expressed as frequency and percentage. The mean of continuous variables was compared between the two groups using an independent *t*-test or the Mann-Whitney U test. Categorical variables were compared using the chi-square test. Inter-assay variability measured the consistency of replicate samples between experiments (HbA1c at different times). The formula for the coefficient of variation is %CV = (standard deviation/mean) × 100. According to guidelines, inter-assay %CVs of less than 15 are generally considered acceptable. Additionally, repeated measures analysis of variance (ANOVA) was used to test changes in HbA1c after therapy in the celiac group. Data analysis was conducted using SPSS version 25.0 (SPSS Inc., USA), and a P-value of < 0.05 was considered significant.

## 4. Results

### 4.1. Participants’ Characteristics

**Figure 1.**
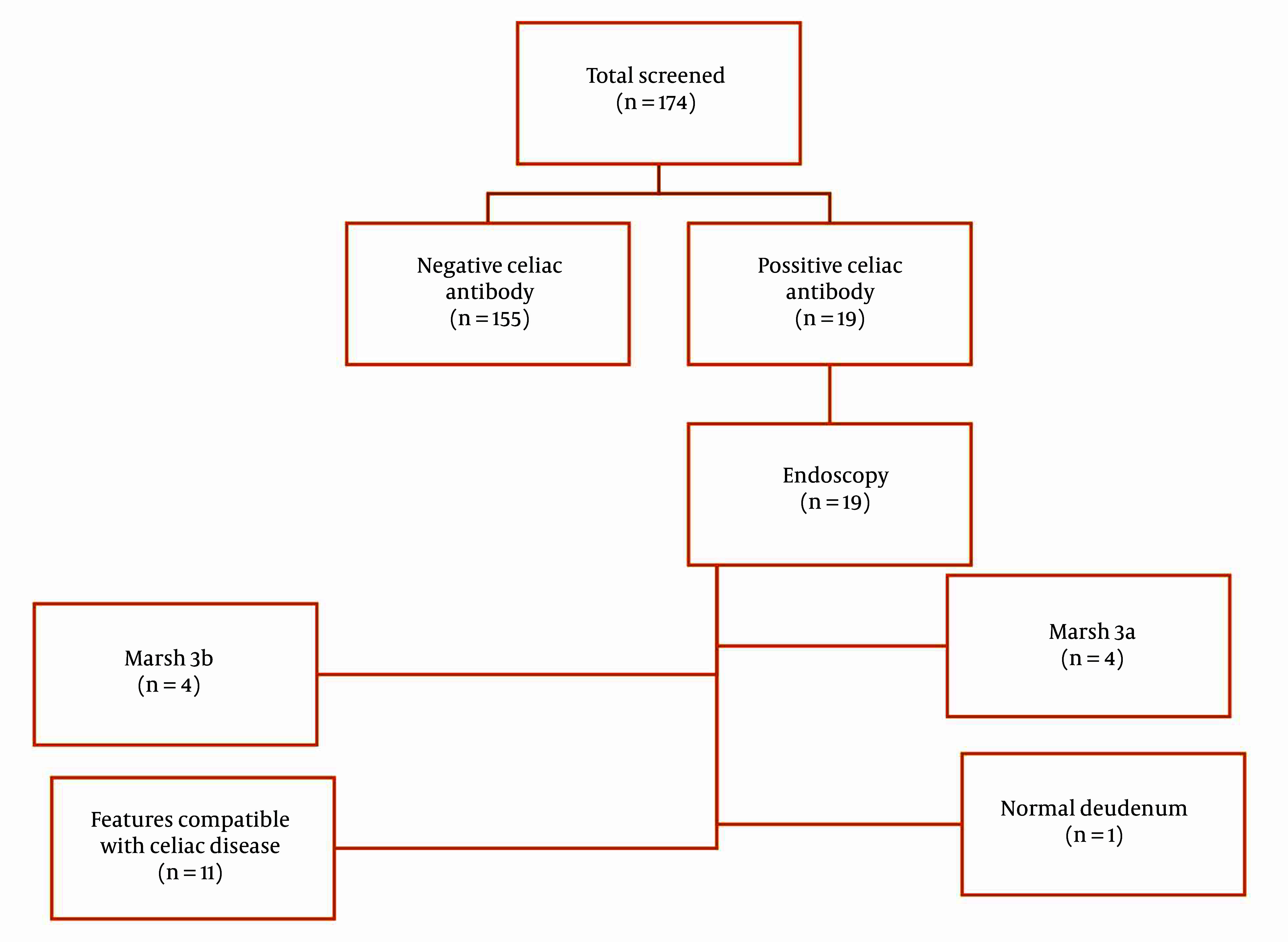
Study flow for screening and diagnosis of celiac disease in patients with type 1 diabetes mellitus

We assessed 174 children with T1DM (81 boys and 93 girls) from Mofid Children’s Hospital. The patients' mean age at the time of sampling was 9.90 ± 3.92 years, and the mean age at diabetes onset was 7.23 ± 3.57 years. The mean percentiles for height, weight, and BMI were 50.12% ± 29.42%, 53.95% ± 29.52%, and 52.25% ± 30.32%, respectively. Among these patients, 18 were seropositive (anti-tTG > 40 U/mL), one patient was persistently positive for anti-tTG at a lower titer (20 - 40 U/mL), and none had IgA deficiency. CD was confirmed by biopsy in 18 out of 19 patients (94.73%). Histological reports showed that 4 patients had Marsh 3A, 4 patients had Marsh 3B, and 10 patients had features compatible with CD (Figure 1). The mean age of celiac onset in the celiac group was 9.90 ± 3.35 years, with a diabetes-celiac diagnosis interval of 3.13 ± 2.25 years. In the celiac group, the mean age at diabetes onset was 7.13 ± 3.20 years. There was no significant difference in the age of diabetes onset between the celiac and non-celiac groups (P = 0.430). None of the patients showed clinical symptoms of CD. The prevalence of subclinical hypothyroidism was not significantly different between the two groups (P = 0.853). Anthropometric indices are provided in Table 1. 

**Table 1.** Celiac and Non-celiac Participant’s Characteristics ^a^

Variables	Celiac; (N = 18)	Non-celiac (N=156)	Total; (N = 174)	P-Value
**Gender **				0.445
Male	7 (38.89)	74 (47.40)	81 (46.60)	
Female	11 (61.11)	82 (52.60)	93 (53.40)	
**Age at the time of sampling (y)**	10.60 ± 3.14	9.81 ± 3.99	9.90 ± 3.92	0.440
**Age at diabetes diagnosis (y)**	7.13 ± 3.20	7.28 ± 3.66	7.23 ± 3.57	0.430
**Age at celiac diagnosis (y)**	9.89 ± 3.30	-	9.89 ± 3.30	N/A
**TTG (U/mL)**	187.01± 119.68	5.72±23.3	25.03 ± 71.89	< 0.001
**Height (cm)**	135.11 ± 19.43	136.25 ± 22.45	136.17 ± 22.11	0.763
**Height percentile **	33.47 ± 29.84	51.93 ± 28.90	50.12 ± 29.40	0.015
**Weight (kg)**	33.15 ± 12.27	35.51 ± 15.85	35.31 ± 15.51	0.744
**Weight percentile**	37.65 ± 34.40	55.72 ± 28.50	53.94 ± 29.50	0.026
**BMI (kg/m** ^**2**^ **)**	17.62 ± 2.87	18.04 ± 3.44	18.0 ± 3.38	0.764
**BMI percentile**	44.23 ± 25.63	53.14 ± 30.73	52.25 ± 30.32	0.245
**Diabetes-celiac diagnosis interval (y)**	3.13 ± 2.25	-	3.13 ± 2.25	N/A
**T4 level**	9.23 ± 1.08	8.98 ± 0.95	9.0 ± 0.96	0.105
**TSH level**	2.67 ± 0.95	2.43 ± 1.02	2.45 ± 1.01	0.459
**Number of clinical hypothyroid patients**	0	0	0	N/A
**Number of subclinical hypothyroid patients (%)**	1 (5.55)	9 (5.77)	10 (5.74)	0.853

Abbreviation: N/A, not applicable; BMI, Body Mass Index

^a^ Values are expressed as mean ± SD or No. (%).

### 4.2. Comparison of Demographic Characteristics in Non-celiac and Celiac Patients

The comparison of anthropometric indices between celiac and non-celiac patients is presented in Table 1. No significant difference was observed between girls and boys in the development of CD (P = 0.445). However, the celiac and non-celiac groups showed significant differences in weight and height percentiles (P = 0.015 and P = 0.026, respectively), while there was no significant difference in BMI (P = 0.764).

We categorized the height percentile into three classes: Tall stature (> 97%), normal (3 - 97%), and short stature (< 3%). No significant differences in height were found between the celiac and non-celiac groups (P = 0.131). The same protocol was applied for weight and BMI: Overweight (> 97%), normal (3 - 97%), and underweight (< 3%). No significant differences were observed between the two groups in terms of weight and BMI (P = 0.225 and P = 0.724, respectively).

### 4.3. Variance of HbA1c in the Celiac Group

**Figure 2.**
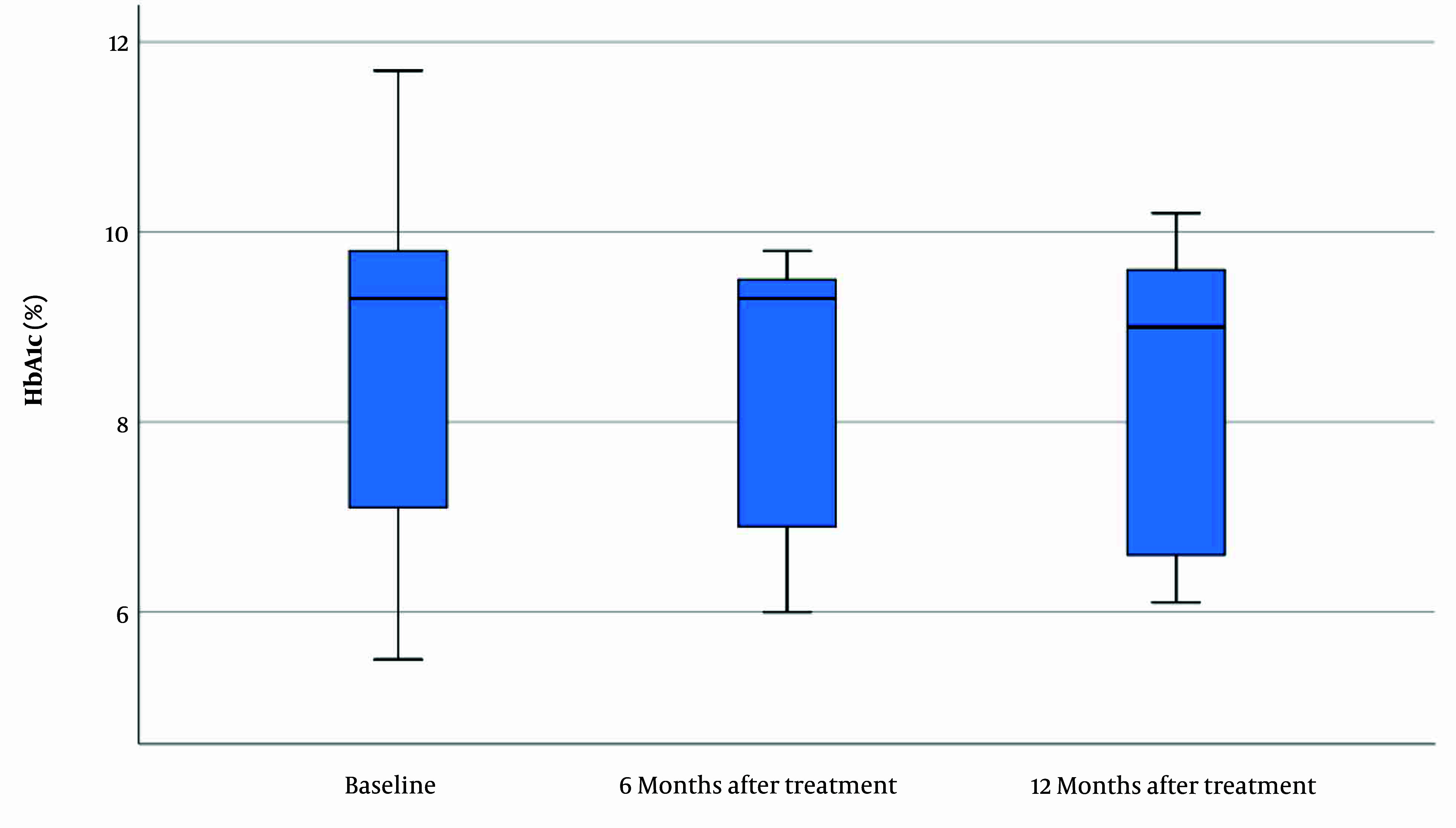
HbA1c levels before and after gluten-free diet (GFD) treatment

The mean HbA1c in the celiac group was 8.61 ± 2.20 (CI = 95%, 5.1 - 12.1) prior to GFD treatment. HbA1c was assessed six and 12 months after the GFD and was 8.32 ± 1.46 (CI = 95%, 6 - 9.8) and 8.37 ± 1.67 (CI = 95%, 6.1 - 10.2), respectively. The mean inter-assay coefficient of variation (CV) for HbA1c was 4.9%. Figure 2 shows that no significant changes were found in HbA1c before and after the therapy (P = 0.501).

## 5. Discussion

In the current study, we explored the prevalence of CD among type 1 diabetic patients and evaluated differences in anthropometric indices between celiac and non-celiac patients. In the celiac group, the variance in HbA1c before and after treatment was also recorded. This study showed that celiac patients had significantly lower height and weight, but their BMI was the same as non-celiac patients. Additionally, a GFD had no effect on HbA1c concentration.

In this group of type 1 diabetic patients, histopathological features compatible with CD were observed in 10.40% of the studied patients. Marsh 3 in biopsy findings was 4.62%, and 5.78% had other pathological findings compatible with CD. The prevalence of definite CD in Iran was reported to be approximately 3% in other studies (1, 29-32). Compared with our results, CD prevalence in type 1 diabetic children is 3.33 times higher than in the non-diabetic patient population. There is no exact age of onset for CD in T1DM patients, but a previous study reported a 5.5-year interval between the onset of type 1 diabetes and CD diagnosis (33). Considering our 2-year follow-up, this may affect the incidence rate reported in this study.

In our study, height and weight percentiles showed significant differences between non-celiac and celiac patients, but BMI was similar in the studied groups. This similarity in BMI may be due to a proportional decrease in both height and weight indices in the same group. Rami et al. (34) evaluated BMI and height standard deviation scores. In contrast to our study, they found that height did not differ between the celiac and non-celiac groups; however, in agreement with our evaluation, patients' BMIs were similar in each studied group. They evaluated patients with silent celiac, which may explain the differences in the results. Oujamaa et al. (35) compared clinical and biological characteristics in type 1 diabetic patients who developed CD with non-CD type 1 diabetic patients. Consistent with our results, CD patients had significantly lower height.

According to the HbA1c measurements in celiac patients, there were no significant changes before and after a GFD. This finding indicates that CD treatment did not have a significant effect on HbA1c levels. There are some controversies over the influence of GFD treatment on metabolic parameters such as HbA1c. The results of Saadah et al. (36) support our study, as they reported no change in HbA1c levels before and after GFD. In a meta-analysis, Burayzat et al. examined changes in HbA1c across six studies and found no significant changes in HbA1c in patients with CD and type 1 diabetes before and after a GFD (37). The prevalence of hypoglycemic episodes, poor glycemic control, and unpredictable glucose levels is higher in diabetic children with CD (38). Hypoglycemic and hyperglycemic episodes occur with a higher probability in patients with coexisting T1DM and CD (39). The mechanism behind unexplained hypoglycemia-hyperglycemia in these children might be due to immune enteropathy or inadequate intake due to gastrointestinal symptoms.

It appears that the use of HbA1c levels as a predictor of glycemic control in coexisting T1DM and CD has limitations. It is suggested to assess glycemic control in diabetic CD patients treated with a GFD using methods such as continuous glucose monitoring (CGM) to determine the duration of target glycemic values.

The present study had some limitations. We did not measure autoantibodies for the diagnosis of type 1 diabetes in patients. Additionally, we did not calculate the daily insulin dosage, daily blood sugar levels, or blood glucose variability.

### 5.1. Conclusions

There is an association between T1DM and CD in our series; however, this association may not be generalizable to all Iranian children due to the non-representative nature of the samples. The rate of CD in type 1 diabetic patients is three times higher than that in the normal population. The anti-tTG antibody successfully diagnoses 94% of celiac cases. Celiac disease patients have lower weight and height compared to non-CD patients, but there is no correlation between BMI and CD. A GFD in CD patients has no effect on HbA1c levels. Patients with T1DM and CD may have higher blood glucose variability, making the interpretation of HbA1c levels challenging.

## Data Availability

The dataset presented in the study is available on request from the corresponding author during submission or after publication.
